# Performance Prediction and Process Optimization of Aging-Resistant Rubber-Modified Asphalt via Enhanced BP Neural Network and Multi-Objective NSGA-II

**DOI:** 10.3390/ma18235292

**Published:** 2025-11-24

**Authors:** Shanwei Li, Shaojie Gao, Jiangtao Fan, Jiupeng Zhang, Yan Li

**Affiliations:** 1 The Key Laboratory of Intelligent Construction and Maintenance of CAAC, Chang’an University, Xi’an 710064, China; lishanwei@chd.edu.cn (S.L.); gaoshaojie@chd.edu.cn (S.G.); jiupengzhang@chd.edu.cn (J.Z.); 2 School of Energy and Architecture, Xi’an Aeronautical University, Xi’an 710077, China; 2020021070@chd.edu.cn; 3 School of Civil Aviation, Northwestern Polytechnical University, Xi’an 710072, China

**Keywords:** rubber asphalt, crested porcupine optimization, dung beetle optimization, SHAP analysis, NSGA-II algorithm

## Abstract

The complex nonlinear interplay between preparation parameters and macroscopic properties poses challenges for predicting the performance of anti-aging rubber asphalt. To address this, two bio-inspired algorithms—Crested Porcupine Optimizer (CPO) and Dung Beetle Optimizer (DBO)—were integrated with a backpropagation (BP) neural network, forming CPO-BP and DBO-BP hybrid models for multi-target prediction. The CPO-BP model demonstrated superior predictive accuracy, significantly outperforming both the standard BP and DBO-BP models, which is attributed to its adaptive global-local optimization mechanism. Shapley additive explanations (SHAP) analysis identified mixing temperature as the most influential factor, with elevated values enhancing rutting resistance but compromising ductility, while moderate temperatures improved aging resistance. Feature interactions indicated synergistic effects between mixing temperature and shear time, and a strong coupling effect between rubber content and temperature on low-temperature performance. Parameter optimization via Non-dominated Sorting Genetic Algorithm II (NSGA-II) further enhanced high–low temperature stability and aging resistance, confirmed by Atomic Force Microscopy (AFM)-based microstructural characterization. The proposed approach provides a robust framework that integrates data-driven prediction and multi-objective optimization for the rational design of high-performance anti-aging rubber asphalt.

## 1. Introduction

Asphalt pavement is widely used in road engineering due to its excellent mechanical properties and construction feasibility [1,2,3,4,5]. However, conventional petroleum asphalt pavements are prone to material aging and performance degradation during long-term service due to combined load and environmental effects [6,7]. In recent years, crumb rubber-modified asphalt (CRMA) derived from waste tires has emerged as an environmentally sustainable alternative. CRMA is produced by high-temperature shearing of base asphalt mixed with crumb rubber particles. During this process, the rubber undergoes devulcanization, absorbs light components from asphalt, and strengthens interfacial bonding, forming a stable cross-linked network [8,9,10,11,12]. This unique microstructure endows CRMA with exceptional elastic recovery [8], crack resistance [13], and low-temperature toughness [14]. However, the inherent multiphase structure of CRMA complicates its aging mechanisms, with critical performance metrics exhibiting pronounced sensitivity to factors such as rubber content, mesh size, anti-aging additive dosage, mixing temperature, and shear duration [15,16,17]. The complex nonlinear relationships among these parameters challenge conventional empirical models, hindering accurate performance prediction and large-scale CRMA application.

Traditional prediction methods in materials science, such as multivariate regression and gray relational analysis, suffer from long experimental cycles, high costs, and limited ability to model nonlinear multi-variable interactions [15,18]. Recent advances in machine learning have introduced novel paradigms for data-driven material modeling [19]. Backpropagation (BP) neural networks, leveraging their nonlinear mapping capabilities, have demonstrated success in asphalt rheological parameter prediction and mixture performance evaluation [19,20]. Zhang et al. achieved high accuracy prediction of needle penetration of aged asphalt by constructing an asphalt binder aging prediction model based on a BP neural network [21]. Luo et al. verified the effectiveness of the BP network in water damage resistance of recycled asphalt mixtures by taking void ratio, number of freeze–thaw cycles, and recycling agent content as input variables [22]. Ma et al. employed a backpropagation neural network to determine the relationship between the asphaltene fraction and the fatigue performance of the binder [23]. Nevertheless, conventional BP neural networks are hindered by slow convergence, entrapment in local minima, and sensitivity to initial conditions—challenges that are exacerbated in high-dimensional, strongly coupled scenarios, where predictive accuracy and generalizability frequently fail to satisfy practical engineering demands.

To address these challenges, hybrid optimization strategies integrating heuristic algorithms have been proposed. Genetic algorithms (GA) and particle swarm optimization (PSO) have been implemented to enhance network initialization, achieving notable improvements [24,25,26]. The PSO-BP neural network model proposed by Zhang et al. was able to accurately predict the damage characteristics of nanoTiO_2_-reinforced concrete after freeze–thaw cycles [27]. Tian applied the PSO-BP model to predict the production of wells in a typical slit-hole reservoir area of the Tahe Oilfield in China and verified it by comparing it with the accuracy of the BP neural network [28]. Nevertheless, as summarized in Supplementary Table S1, these conventional hybrid models possess inherent limitations. They often struggle with premature convergence in complex, high-dimensional parameter spaces and are predominantly applied to single performance indicators. In contrast, the engineering application of CRMA necessitates the synergistic optimization of multiple, often conflicting targets (e.g., high-temperature stability vs. low-temperature ductility). The strong heterogeneity in multi-objective outputs substantially increases modeling complexity. Crucially, no prior research has established an interpretable, multi-target prediction and optimization framework specifically for anti-aging CRMA systems—a critical knowledge gap this study aims to bridge.

In recent years, novel bio-inspired algorithms such as the Crested Porcupine Optimizer (CPO) and Dung Beetle Optimizer (DBO) have provided new approaches to address the aforementioned issues. The CPO algorithm simulates the biological behaviors of crested porcupines in nature, including foraging, predator avoidance, and survival strategies, while ensuring population diversity and enhancing convergence speed [29,30]. It demonstrates particular suitability for multi-parameter systems with non-uniform distribution characteristics. The DBO algorithm establishes multimodal search strategies in high-dimensional spaces by simulating dung beetle behaviors such as ball-rolling, reproduction, and foraging [31,32]. Its adaptive segmented optimization mechanism significantly improves global convergence efficiency. However, current research shows no reported applications of these algorithms in multi-objective performance prediction for rubberized asphalt, with insufficient exploration of cross-scale correlation mechanisms between preparation parameters and performance indicators.

Based on this, this study proposes a comprehensive framework that integrates data-driven modeling with multi-objective optimization for the rational design of anti-aging rubber asphalt. The work unfolds across three interconnected phases to address the complex mapping relationships between preparation parameters and multi-objective performance. First, dual-optimized neural network models (CPO-BP and DBO-BP) are developed, where the CPO and DBO are employed to enhance the convergence and accuracy of the foundational BP neural network by adeptly navigating the high-dimensional weight space. The models are rigorously trained and validated using a hold-out test set and 5-fold cross-validation, with multiple independent runs ensuring computational repeatability and statistical robustness. Second, to transcend “black-box” prediction, Shapley additive explanations (SHAP) is leveraged for post hoc interpretability and global sensitivity analysis, quantifying the contribution of each preparation parameter and revealing critical nonlinear interaction effects. Finally, the validated models are coupled with the Non-dominated Sorting Genetic Algorithm II (NSGA-II) for multi-objective optimization, identifying the Pareto-optimal frontier that trades off rutting resistance, low-temperature ductility, and aging resistance. The final optimal solution is selected from this frontier, providing a principled and intelligent decision-making support system for formulating high-performance, eco-friendly road materials.

## 2. Materials and Methods

### 2.1. Materials and Database Acquisition

#### 2.1.1. Materials

The asphalt binder is 70# base asphalt, with its technical parameters presented in Supplementary Table S2.

The rubber powder used in this study was processed from waste tires, with a particle size range of 40–100 mesh. Its technical parameters are listed in Supplementary Table S3.

The anti-aging agent used in this study is a phosphite antioxidant with the molecular formula C_42_H_6_O_3_P, which mitigates asphalt aging by decomposing hydroperoxides generated during the aging process of modified asphalt.

**Figure 1:**
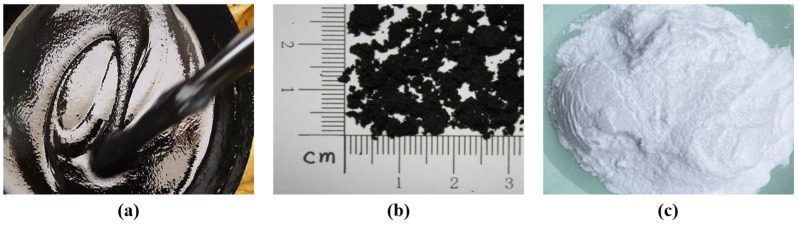
Raw materials: (**a**) asphalt; (**b**) rubber powder; (**c**) anti-aging agent.

Photographs of the raw materials are presented in Figure 1.

#### 2.1.2. Experimental Methods

(1) Sample Preparation

The CRMA was prepared via the wet process using a high-temperature shearing method. The base asphalt was first heated to a fluid state. Subsequently, crumb rubber powder and the phosphite antioxidant were gradually added. The mixture was then subjected to high-speed shearing using a mechanical shear mixer at 5000 rpm to ensure homogeneous dispersion. The five key preparation parameters—rubber powder dosage (RPD: 10–30%), rubber powder mesh size (RPM: 40–100 mesh), anti-aging agent dosage (AD: 0.3–2.0%), mixing temperature (MT: 155–215 °C), and shearing time (ST: 25–135 min)—were systematically varied to construct the experimental database.

(2) Performance Tests

Rutting Factor: The high-temperature rutting resistance, represented by the rutting factor at 60 °C, was determined using a Dynamic Shear Rheometer (DSR) according to ASTM D7175 [33]. The test was conducted in a controlled-stress mode with a frequency of 10 rad/s and within the linear viscoelastic region.

Ductility: The low-temperature crack resistance, indicated by ductility at 5 °C, was measured via a ductilometer test as per ASTM D113 [34].

Residual penetration ratio: To simulate short-term aging and evaluate the aging resistance, the prepared CRMA samples underwent the Rolling Thin Film Oven Test (RTFOT) following ASTM D2872 [35]. The aging resistance was quantified by the Residual Penetration Ratio, which was calculated as the ratio of the penetration value at 25 °C (ASTM D5 [36]) after RTFOT aging to the penetration value of the original unaged sample.

Microstructural Characterization: Atomic Force Microscopy (AFM) was employed to investigate the micro-morphology of selected samples. Experiments were conducted using Bruker Dimension Icon (Bruker, Bileric, MA, USA) in tapping mode under ambient conditions. Samples were prepared by hot-casting a drop of binder onto a glass substrate. The obtained images were analyzed to extract surface roughness parameters, linking the microscopic structure to macroscopic performance.

#### 2.1.3. Database Acquisition

The experimental dataset for this study was systematically generated through a controlled laboratory investigation focusing on rubber-modified asphalt properties. Five key process parameters were selected as feature variables: rubber powder dosage (RPD), rubber powder mesh size (RPM), anti-aging agent dosage (AD), mixing temperature (MT), and shearing time (ST).

**Figure 2:**
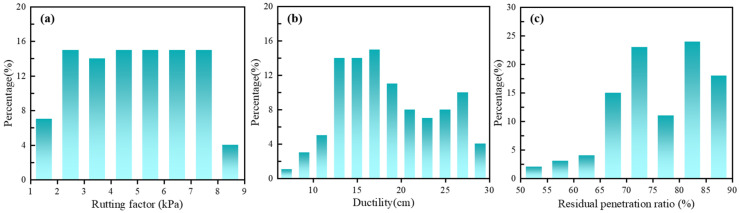
The distribution characteristics of the three target variables: (**a**) Rutting factor; (**b**) Ductility; (**c**) Residual penetration ratio.

This study evaluated 100 parameter combinations and obtained 100 sample data points, with each datum representing the average value from three repeated experimental trials. A representative subset of the experimental data and the comprehensive statistical analysis results of the complete dataset are provided in Supplementary Tables S4 and S5, respectively. The distribution characteristics of the three target variables (Rutting factor, Ductility, and Residual penetration ratio) are visualized through histogram representations in Figure 2.

**Figure 3:**
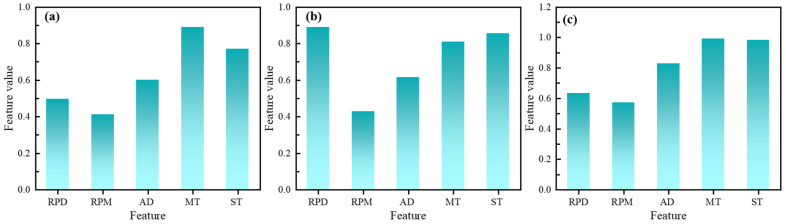
The feature importance of the three target variables: (**a**) Rutting factor; (**b**) Ductility; (**c**) Residual penetration ratio.

A random forest regression algorithm was used to quantify the feature importance of five preparation parameters of rubber asphalt in the database, aiming at initially revealing the mechanism of differential influence of different process variables on multi-objective performance, as shown in Figure 3. Based on 100 sets of experimental data, a random forest model was constructed for rutting factor, low-temperature ductility and residual penetration ratio to assess the contribution of each variable. The key control parameters of rutting factor are mixing temperature and shear time, the reduction in asphalt viscosity under high temperature promotes the swelling of rubber powder, and sufficient shear time ensures the formation of three-dimensional cross-linking network, which synergistically enhances the high-temperature resistance to deformation [37,38]. The low-temperature elongation is mainly dominated by the rubber powder dosage and the shear time, and the increase in the rubber powder dosage inhibits low-temperature brittle cracking through the increase in flexibility of the material, but it is necessary to match with the appropriate shear time to avoid excessive degradation [39,40]. The dominant factors of anti-aging performance are shear time and anti-aging agent dosage, where long shear time can refine the dispersion of rubber powder to reduce the aging interface defects, and the anti-aging agent dosage can slow down the oxidation process through the effect of free radical trapping. It is worth noting that the mesh size of rubber powder has the weakest effect on all the performance indexes, which may be attributed to the fact that the change in mesh size is partially offset by the effect of particle size reconstruction during the shearing process.

### 2.2. Machine Learning Model Construction

#### 2.2.1. BP Neural Network and Optimization Algorithms

(1) BP Neural Network

The BP neural network is a multilayer feedforward network based on gradient descent, whose core mechanism lies in dynamically adjusting weights and biases through error backpropagation [41]. The network architecture comprises an input layer, hidden layer(s), and output layer, with data processed through forward propagation as follows:

The output of each node from the input layer to the implicit layer is shown in Equation (1):

(1) $$h_{j}=f\left(\sum_{i=1}^{n}w_{ij}x_{i}+b_{j}\right)$$

where *h_j_* denotes the *j*-th node of the hidden layer, *w_ij_* is the weight of the input layer to the hidden layer, *b_j_* is the bias, and *f* is chosen as a Sigmoid function, shown in Equation (2):

(2) $$f\left(z\right)=\frac{1}{1+e^{-z}}$$

The output of each node from the implicit layer to the output layer is shown in Equation (3):

(3) $$y_{k}=f\left(\sum_{j=1}^{m}w_{jk}h_{j}+b_{k}\right)$$

where *y_k_* denotes the *j*-th node of the hidden layer, *w_jk_* is the weight of the hidden layer to the output layer, *h_j_* is the bias.

Then, error backpropagation is performed, where the propagated error is the variance of the desired output from the actual output, and the loss function is defined as Equation (4):

(4) $$E=\frac{1}{2}\sum_{k=1}^{p}{\left(y_{k}-t_{k}\right)}^{2}$$

where *y_k_* denotes the expected value of the *k*-th node of the output layer; *t_k_* denotes the actual output of the *k*-th node of the output layer.

(2) Crested Porcupine Optimization Algorithm

The CPO algorithm was employed to optimize the initial weights and biases of the BP network, addressing its limitations of slow convergence and susceptibility to local minima. The algorithm’s multi-modal defense strategies enable dynamic balance between global exploration and local exploitation during the optimization process.

The CPO algorithm is a novel metaheuristic algorithm that has both exploration and exploitation mechanisms by simulating the defensive behaviors of crested porcupines in evading predators [42]. In the exploration phase, the crested porcupine initiates defense based on the location of the predator, and when the predator is far away, the crested porcupine herd has two defense strategies, a visual strategy and an auditory strategy. In the exploitation phase, when the predator approaches to stimulate the defensive behavior of the crested porcupine herd, the crested porcupine population has two defensive strategies, the scent strategy and the attack strategy [43,44].

At the beginning, crested porcupines were randomly assigned to a specific location and waited for a predator to appear. The initial position is:

(5) $$X_{i}=L+r\cdot \left(U-L\right)$$

where *N* is the number of candidate solutions, *X*_i_ is the ith candidate solution, *L* is the upper boundary of the search, *U* is the lower boundary of the search, and *r* is an initialization vector ranging from 0 to 1.

When the crested porcupine realizes that a predator is present, it raises its wings so that the predator has two choices: either approach or leave. If the predator chooses to approach, the distance between them decreases, which increases the algorithm’s convergence rate. If the predator chooses to stay away, the distance increases and the algorithm maximizes the global search capability and avoids local optima, which is modeled as:

(6) $$X_{i}^{t+1}=X_{i}^{t}+\tau_{1}\cdot \left(2\times \tau_{2}\cdot X_{best}^{t}-Y_{i}^{t}\right)$$

where $X_{best}^{t}$ is the optimal solution, $Y_{i}^{t}$ is the position of the predator, $\tau_{1}$ is a random number generated from a normal distribution, and $\tau_{2}$ is a random value range interval of [0, 1].

In an acoustic defense strategy, crested porcupines repel predators by making sounds. The closer the predator moves, the louder the sound the crested porcupine makes as a way to scare off the predator, which is modeled as:

(7) $$X_{i}^{\begin{array}{c} t+1 \end{array}}=\left(1-\vec{U_{1}}\right)\cdot X_{i}^{t}+\vec{U_{1}}\cdot \left(Y_{i}+\tau_{3}\cdot \left(X_{r1}^{t}-X_{r2}^{t}\right)\right)$$

where *r*_1_ and *r*_2_ are two random integers between [1, N], $\tau_{3}$ is a random number in the range [0, 1], $\vec{U_{1}}$ is a randomly generated binary vector of [0, 1].

To prevent predators from approaching, crested porcupines secrete a foul scent during the scent defense strategy phase, which drives away predators. Expressed as:

(8) $$X_{i}^{\begin{array}{c} t+1 \end{array}}=\left(1-\vec{U_{1}}\right)\cdot X_{i}^{t}+\vec{U_{1}}\cdot \left(X_{r1}^{t}+S_{i}^{t}\cdot \left(X_{r2}^{t}-X_{r3}^{t}\right)-\tau_{3}\cdot \delta \cdot \gamma_{t}\cdot S_{i}^{t}\right)$$

where *r*_3_ is a random value in the range of [1,n]; *δ* is the search direction parameter, $\gamma_{t}$ is the defense factor, and $S_{i}^{t}$ is the diffusion factor, which takes the value in the range of 0.3 to 2.6.

In terms of attack strategy, the crested porcupine deploys its short, thick spines, which it uses to attack a predator as it approaches, modeled as follows:

(9) $$X_{i}^{\begin{array}{c} t+1 \end{array}}=X_{best}^{t}+\left(\alpha \left(1-\tau_{4}\right)+\tau_{4}\right)\cdot \left(\delta \cdot X_{best}^{t}-X_{i}^{t}\right)-\tau_{5}\cdot \delta \cdot \gamma_{t}\cdot F_{i}^{t}$$

(10) $$F_{i}^{t}=\tau_{6}\cdot m_{i}\cdot \left(\nu_{i}^{t+1}-\upsilon_{i}^{t}\right)$$

where α is the convergence speed factor; $\tau_{3},\tau_{4},\tau_{5}$ are random numbers within [0, 1]; $F_{i}^{t}$ is the average individual force affecting the *i*-th predator, provided by the inelastic collision law; $m_{i}$ is the mass of the *i*-th predator at the time of the iteration; $\upsilon_{i}^{t+1}$ is the final velocity of individual at the next iteration t + 1; $\nu_{i}^{t}$ is the initial velocity of individual at iteration *t*.

(3) Dung Beetle Optimization Algorithm

The DBO is a bio-inspired metaheuristic algorithm that simulates four distinct behavioral phases of dung beetles—ball rolling, dancing, breeding, and foraging—to balance global exploration and local exploitation in solving complex nonlinear optimization problems [45,46,47]. Its core innovation lies in decomposing the optimization process into four mathematically modeled stages, each governed by specific evolutionary strategies. Similarly to CPO, the DBO enhances BP network training through its unique rolling, breeding, and foraging mechanisms, providing an alternative approach to navigate the complex weight space and improve prediction accuracy.

In the ball rolling phase, individuals propel their dung balls toward the solar azimuth (analogous to gradient descent direction) with stochastic angular deviations and adaptive step sizes:

(11) $$X_{i}^{t+1}=X_{i}^{t}+\theta \cdot L\cdot \left(X_{best}^{t}-X_{i}^{t}\right)$$

where $X_{best}^{t}$ is global best solution; θ is random deflection angle, modeling the uncertainty of the rolling path; *L* is the rolling step size, usually the distance of the current individual from the optimal solution.

In the dancing phase, beetles perform stochastic helical dances to perturb rolling trajectories, preventing premature convergence:

(12) $$X_{i}^{t+1}=X_{i}^{t}+\tan\left(\phi \right)\cdot \|X_{i}^{t}-X_{best}^{t}\|$$

where ϕ is the random perturbation angle, obeying a uniform distribution; $\tan\left(\phi \right)$ generates positive and negative perturbations, balancing the direction of exploration.

In the breeding phase, beetles generate offspring in the vicinity of the optimal solution, with the radius R gradually shrinking to enable a coarse-to-fine localized search:

(13) $$X_{egg}^{t}=X_{best}^{t}+R\cdot \left(X_{ub}-X_{lb}\right)\cdot \Gamma \left(0,1\right)$$

where *R* is the reproduction radius, which decays with the number of iterations; $X_{ub},X_{lb}$ are the upper and lower bounds of the solution space; $\Gamma \left(0,1\right)$ is a standard normally distributed random number, which controls the distribution of offspring.

In the foraging phase, the offspring individuals gradually converge to the global optimal solution to accelerate the local convergence.

(14) $$X_{l}^{t+1}=X_{l}^{t}+\sigma \cdot \left(X_{best}^{t}-X_{l}^{t}\right)$$

where $X_{l}^{t}$ is the position of the offspring individual; σ is the learning rate, which controls the convergence rate, σ=0.5∼1.0.

#### 2.2.2. Construction of BP Neural Network Models

**Figure 4:**
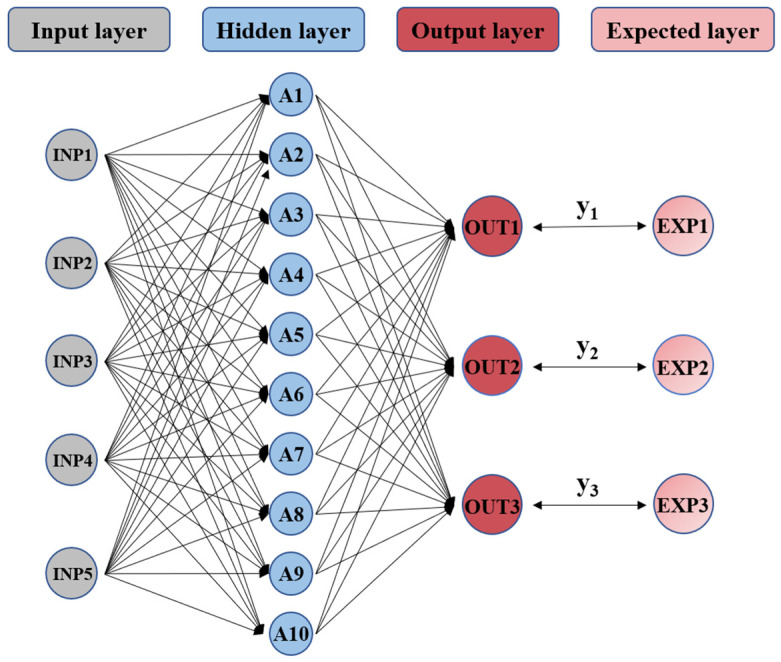
Topological structure of BP neural network.

**Figure 5:**
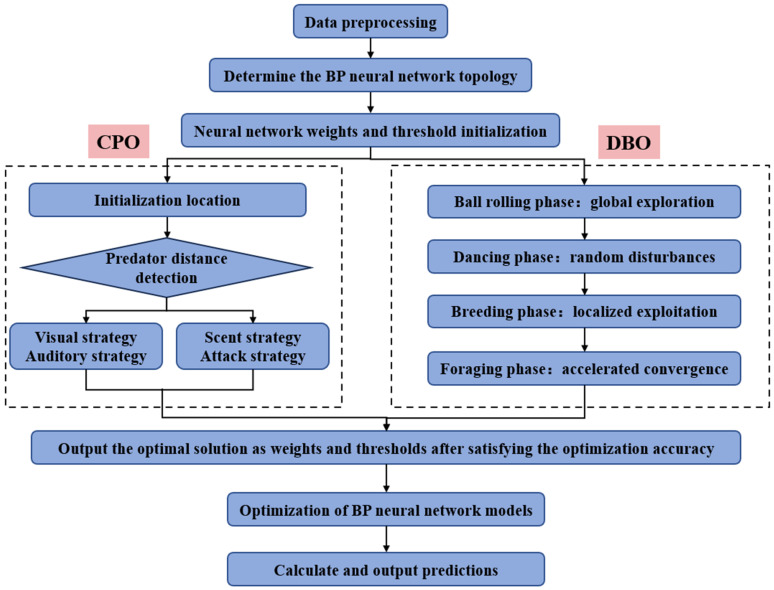
Flowchart of optimization model construction.

Taking RPD, RPM, AD, MT and ST as inputs and Rutting factor, Ductility and Residual penetration ratio as outputs, all five input features were normalized to [0, 1] range using min-max scaling prior to model training. No categorical variables required special treatment as all inputs were continuous numerical parameters. The BP neural network with a neural network topology of 5-10-3 was established, and the structure is shown in Figure 4. Then, the BP model is optimized using CPO and DBO to obtain two optimized models, CPO-BP and DBO-BP, and the construction process is shown in Figure 5. Both the CPO and DBO were configured with a population size of 50 and a maximum of 500 iterations, employing a convergence tolerance of 1 × 10^−6^ over 50 consecutive iterations as the stopping criterion.

All test databases were divided into training and test sets with a ratio of 7:3. To ensure robust hyperparameter tuning and mitigate overfitting, a 5-fold cross-validation strategy was implemented on the training set. During this process, the training set was partitioned into five equal subsets, with four subsets used for training and the remaining one for validation in each iteration. This procedure was repeated five times such that each subset served as the validation set exactly once. The final hyperparameter configuration was selected based on the average performance across all five folds. The model corresponding to the optimal configuration was then retrained on the entire training set, and the test set was used to evaluate the prediction accuracy and generalization ability of the models.

In order to evaluate the applicability of the three models, this study adopts four statistical indicators to comprehensively evaluate the model accuracy. To ensure robust performance estimation, the reported results are based on the average metrics obtained through a 10-fold cross-validation procedure. The mean absolute error (MAE) reflects the average absolute deviation of the predicted value from the true value, which measures the intuitive level of the error. The root mean square error (RMSE) amplifies the weight of larger errors through squaring operation, which is sensitive to identifying the influence of outliers. The mean Absolute Percentage Error (MAPE) quantifies the relative error in percentage form, which is suitable for the horizontal comparison of different quantitative indicators. Coefficient of Determination (*R*^2^) reflects the model’s ability to capture data variability, with values closer to 1 indicating a better fit between predicted and observed results. Among them, MAE and RMSE focus on absolute error analysis, MAPE highlights the relative error characteristics, and *R*^2^ assesses the overall performance in terms of variance explanation. The combination of the four metrics can comprehensively analyze the strengths and weaknesses of the model in terms of accuracy, stability and generalization ability. The formulas for these metrics are as follows:

(15) $$MAE=\frac{1}{n}\sum_{i=1}^{n}\left|y_{i}-{\hat{y}}_{i}\right|$$

(16) $$RMSE=\sqrt{\frac{1}{n}\sum_{i=1}^{n}{\left(y_{i}-{\hat{y}}_{i}\right)}^{2}}$$

(17) $$MAPE=\frac{1}{n}\sum_{i=1}^{n}\left|\frac{y_{i}-{\hat{y}}_{i}}{y_{i}}\right|\times 100\%$$

(18) $$R^{2}=1-\frac{\sum_{i=1}^{n}{\left(y_{i}-{\hat{y}}_{i}\right)}^{2}}{\sum_{i=1}^{n}{\left(y_{i}-\bar{y}\right)}^{2}}$$

where $y_{i}$ represents the actual values, ${\hat{y}}_{i}$ represents the predicted values, $\bar{y}$ is the mean of the actual values, *n* is the total number of samples.

### 2.3. Shapley Additive Explanations

Machine learning models often function as “black boxes”, where the relationship between input features and predictive outcomes remains opaque. To address this limitation, SHAP analysis was employed to interpret the contribution of input features on model predictions. This approach not only quantifies the individual impact of each feature but also reveals potential synergistic interactions between variables.

This study calculated and visualized SHAP values and their interaction values using the shapviz package in the RStudio platform (version 2025.05.1+513). The underlying principle employs the Kernel SHAP method, which is an extension of classical Shapley value estimation that uses specially weighted local linear regression to approximate SHAP values. The calculation is as follows:

(19) $$\varphi_{i}=\sum \left(S\subseteq N\left\{i\right\}\right)\frac{\left[\left|S\right|!\left(M-\left|S\right|-1\right)!\right]}{M!}\left(f(S\cup \left\{i\right\})-f\left(S\right)\right)$$

where $\varphi_{i}$ represents the Shapley value for feature *i*, quantifying its marginal contribution to the model prediction; *N* is the complete set of all features; *S* is a subset of features excluding *I*; *M* is the total number of features.

The formula for calculating the SHAP interaction effect is as follows:

(20) $$\varphi_{i,j}=\sum \left(S\subseteq N\left\{i,j\right\}\right)\frac{\left[\left|S\right|!\left(M-\left|S\right|-2\right)!\right]}{2\left(M-1\right)!}\Delta \left(i,j\right)\left(S\right)$$

(21) $$\Delta \left(i,j\right)\left(S\right)=f\left(S\cup \left\{i,j\right\}\right)-f\left(S\cup \left\{i\right\}\right)-f\left(S\cup \left\{j\right\}\right)+f\left(S\right)$$

where $\varphi_{i,j}$ represents the interaction value between features *i* and *j*; $\Delta \left(i,j\right)\left(S\right)$ measures the pure interaction effect.

## 3. Results and Discussion

### 3.1. Neural Network Model Training, Testing and Evaluation

**Figure 6:**
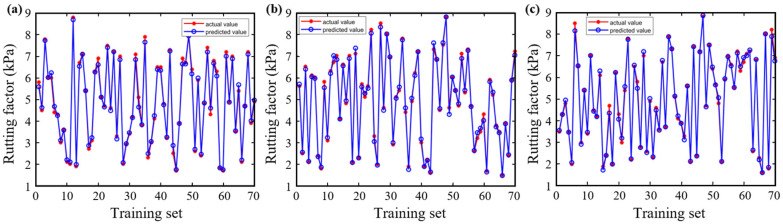
Training of three models for rutting factor: (**a**) BP; (**b**) CPO-BP; (**c**) DBO-BP.

**Figure 7:**
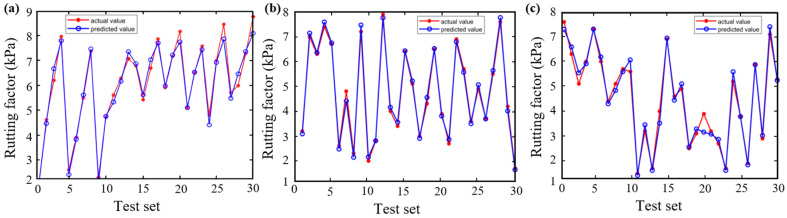
Testing of three models for rutting factor: (**a**) BP; (**b**) CPO-BP; (**c**) DBO-BP.

**Figure 8:**
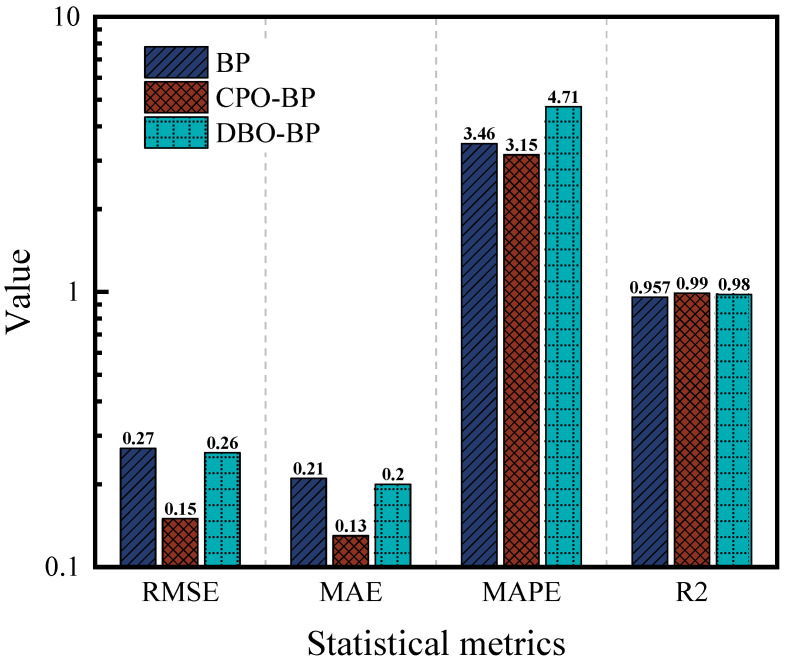
Comparison of statistical metrics of the three models.

Figure 6 and Figure 7 demonstrate the predictive performance of the BP, CPO-BP, and DBO-BP models for the rutting factor on the training and testing sets, respectively, while Figure 8 compares the statistical accuracy metrics of the three models on the testing set. The training set results show that the predicted curves of all three models closely align with the actual values, with *R*^2^ exceeding 0.95, indicating strong nonlinear fitting capabilities. However, significant differences emerge in the testing set predictions: although the BP and DBO-BP models perform well in most regions, they exhibit multiple outliers near local peak areas. In contrast, the CPO-BP model achieves a mean absolute error (MAE) of 0.13 and the highest *R*^2^ (0.99) among the three models, with its trendline accurately tracking inflection points of the actual curve. Compared to the BP model, the CPO-BP model reduces MAE, RMSE, and MAPE by 44.4%, 38.1%, and 9.0%, respectively. The observed discrepancy may be attributed to the BP model’s random weight initialization, which predisposes it to local minima and undermines its ability to generalize in the presence of complex, high-dimensional feature coupling. While the DBO-BP model enhances global search through its ball-rolling mechanism, its fixed-radius contraction strategy during the breeding phase struggles to handle discrete parameters, leading to suboptimal weight-space exploration. The CPO-BP model leverages the multi-modal defense strategies inspired by crested porcupines’ predator evasion dynamics to dynamically balance global search breadth and local refinement depth through distance-dependent strategy switching, effectively resolving the conflict between premature convergence and optimization stagnation in high-dimensional asphalt performance prediction tasks, thereby significantly mitigating overfitting risks. The results corroborate Wang’s findings on the predictive efficacy of CPO for composite materials, thereby reinforcing its unique strengths in addressing the challenges of complex material modeling [48].

**Figure 9:**
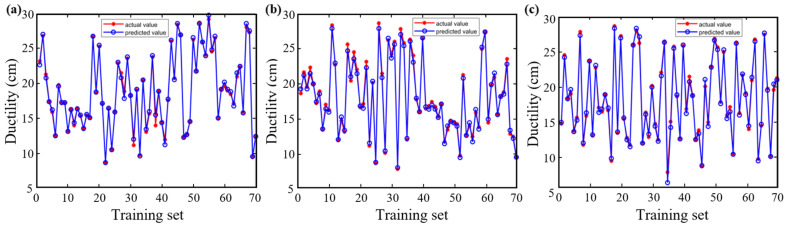
Training of three models for ductility: (**a**) BP; (**b**) CPO-BP; (**c**) DBO-BP.

**Figure 10:**
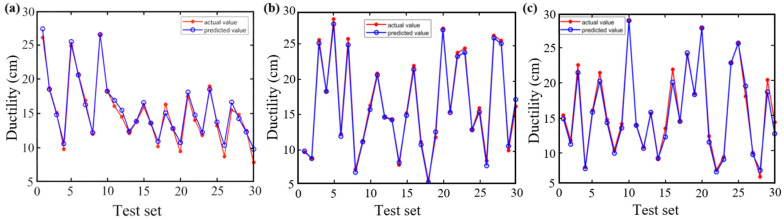
Testing of three models for ductility: (**a**) BP; (**b**) CPO-BP; (**c**) DBO-BP.

**Figure 11:**
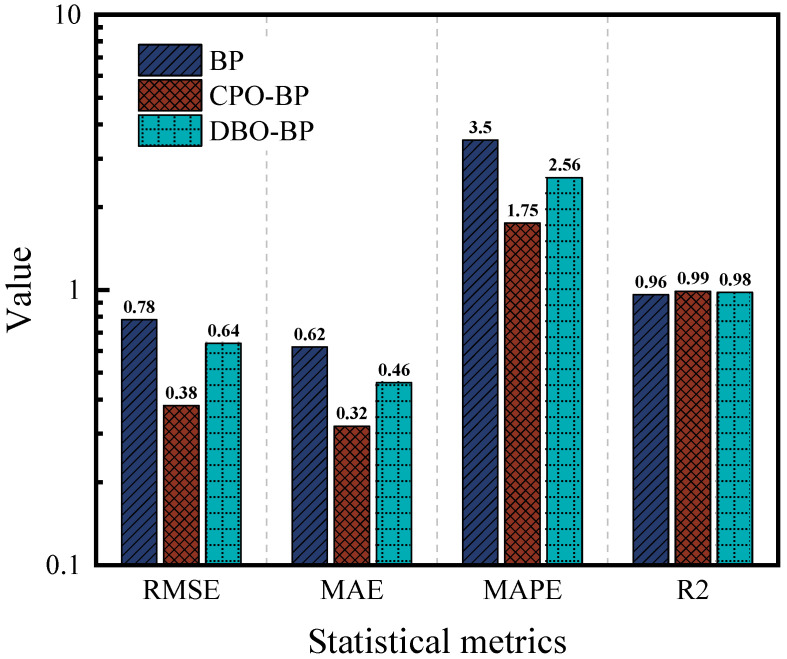
Comparison of statistical metrics of the three models.

Figure 9 and Figure 10 illustrate the predictive performance of the BP, CPO-BP, and DBO-BP models for ductility on the training and testing sets, respectively, while Figure 11 quantifies their performance differences using statistical metrics. The results show that the predicted curves closely align with actual values on the training set, but significant accuracy divergence occurs on the testing set: the BP model shows significant overestimation in the low-temperature delay interval, the DBO-BP model improves over BP, but there are still multiple anomalous fluctuation points, and the CPO-BP demonstrates excellent prediction consistency, with a MAE of only 0.32, an *R*^2^ of as high as 0.99, and a MAPE (1.75%) is significantly lower than BP (3.5%) and DBO-BP (2.56%). Specifically, the MAE, RMSE, and MAPE of CPO-BP were 51.3%, 48.4%, and 50.0% lower than those of the BP model, respectively, verifying its robustness advantage. These results align with the rutting factor predictions, further validating the generalizability and practical applicability of CPO-BP in multi-objective performance prediction for rubberized asphalt.

**Figure 12:**
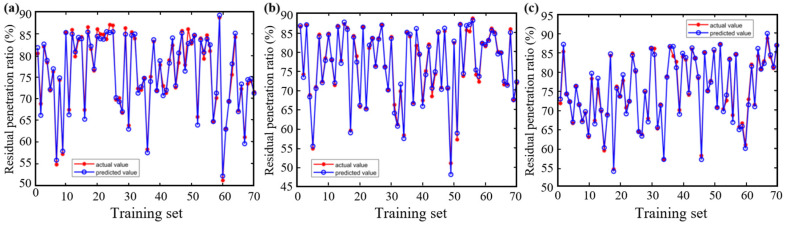
Training of three models for residual penetration ratio: (**a**) BP; (**b**) CPO-BP; (**c**) DBO-BP.

**Figure 13:**
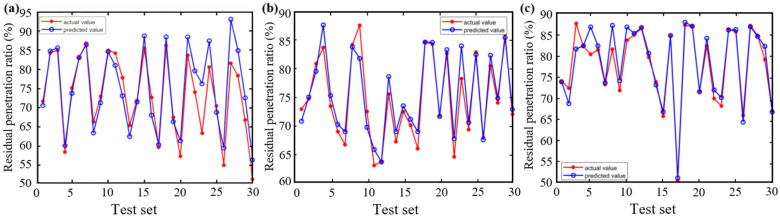
Testing of three models for residual penetration ratio: (**a**) BP; (**b**) CPO-BP; (**c**) DBO-BP.

**Figure 14:**
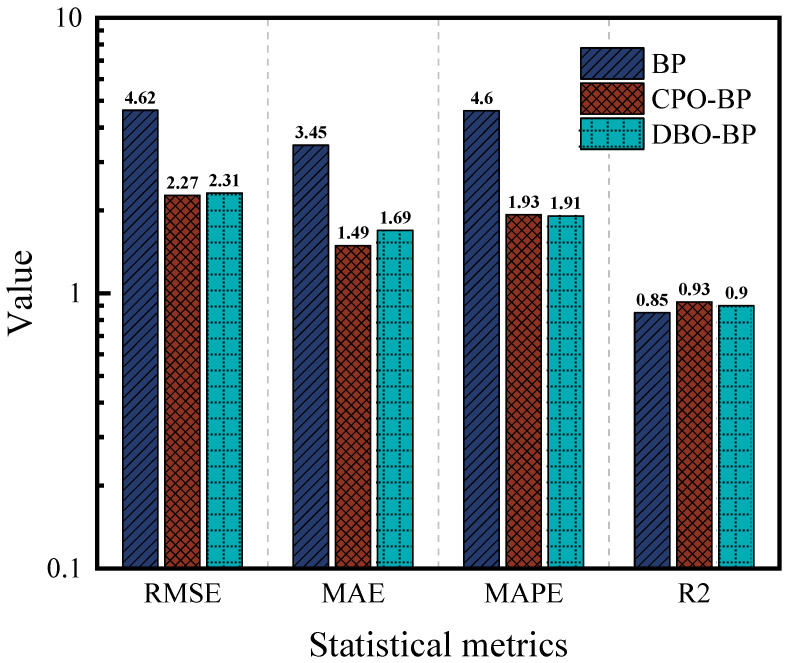
Comparison of statistical metrics of the three models.

Figure 12 and Figure 13 compare the predictive performance of the three models for the residual penetration ratio, while Figure 14 summarizes their statistical metrics. The BP model shows significant deviation in the training set (R^2^ = 0.94), and the performance in the test set is further deteriorated (R^2^ = 0.85), and the prediction accuracies are significantly lower than those of the CPO-BP and the DBO-BP. Although the CPO-BP and the DBO-BP perform well in the training set, their prediction accuracies also deteriorate in the test set, and their R^2^ drops to 0.93 and 0.9, respectively. The main reason for the performance fluctuation is that the nonlinear aging mechanism of the residual needle-entry ratio is affected by the strong coupling of multiple factors, and the insufficient coverage of the training data on the causative conditions leads to the limitation of the model’s generalization ability. In contrast, CPO-BP enhances the weight space traversal through chaotic population initialization, and still maintains the optimal prediction stability.

In addition, the performance prediction models of modified asphalt from similar studies were also compared with the model proposed in this study, and the results are shown in Supplementary Table S6. Compared with the CPO-BP model proposed in this paper, the RMSE of the models are higher and the *R*^2^ values are lower, except for the SVM model in reference [47]. This explains to some extent the reliability and accuracy of the CPO-BP model for prediction in high dimensional and strongly coupled datasets.

### 3.2. Interpretability Analysis Based on SHAP

**Figure 15:**
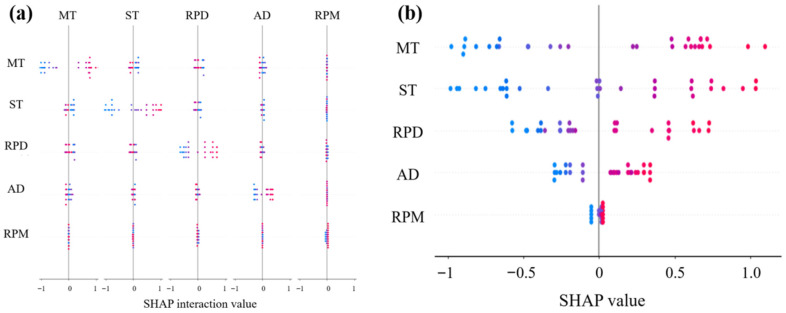
SHAP-based interpretability analysis for rutting factor prediction: (**a**) Interaction influence diagram; (**b**) SHAP summary plot.

**Figure 16:**
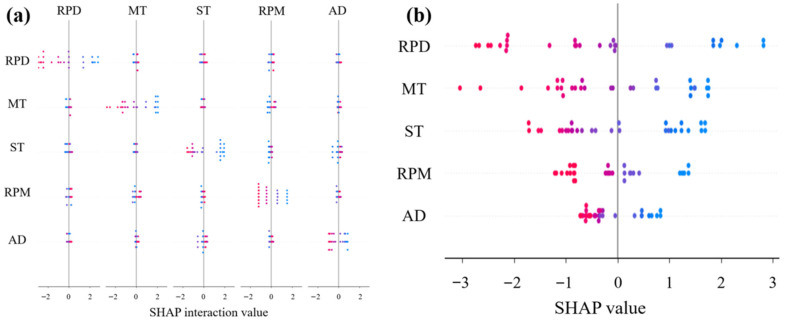
SHAP-based interpretability analysis for ductility: (**a**) Interaction influence diagram; (**b**) SHAP summary plot.

**Figure 17:**
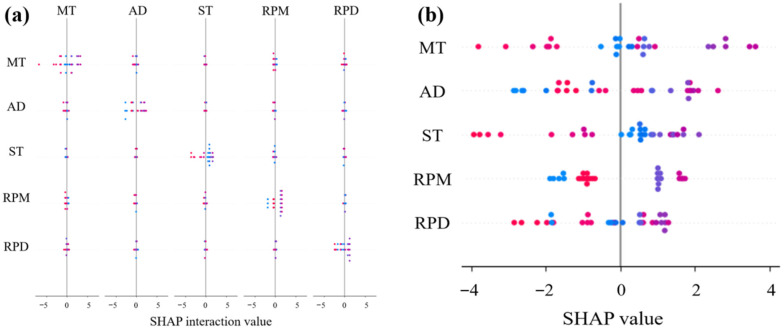
SHAP-based interpretability analysis for residual penetration ratio: (**a**) Interaction influence diagram; (**b**) SHAP summary plot.

The interaction SHAP plots (Figure 15, Figure 16 and Figure 17) demonstrate that mixing temperature consistently exhibits synergistic effects with other parameters across all predictive targets. Specifically, its interaction with shear time significantly influenced rutting factor predictions, while coupling with rubber powder dosage showed notable effects on ductility. However, residual penetration ratio displayed relatively weaker feature interactions compared to other performance indicators. These findings suggest that mixing temperature serves as a pivotal parameter governing the compound effects in rubber asphalt preparation.

The SHAP summary plots (Figure 15b, Figure 16b and Figure 17b) quantitatively verify the directional impacts of key parameters through their distinct value distributions. In Figure 15b, the concentration of high MT values on the positive SHAP axis, colored red, physically corresponds to enhanced rubber devulcanization and crosslinking that improves rutting resistance. Conversely, Figure 16b shows low MT values clustered on the positive SHAP side (blue zone), indicating better preservation of rubber’s elastic networks crucial for low-temperature flexibility. The bimodal distribution in Figure 17b demonstrates that moderate MT values (175–185 °C) maximize aging resistance by balancing anti-aging agent dispersion against oxidative degradation, with both low and high temperature extremes reducing the residual penetration ratio.

**Figure 18:**
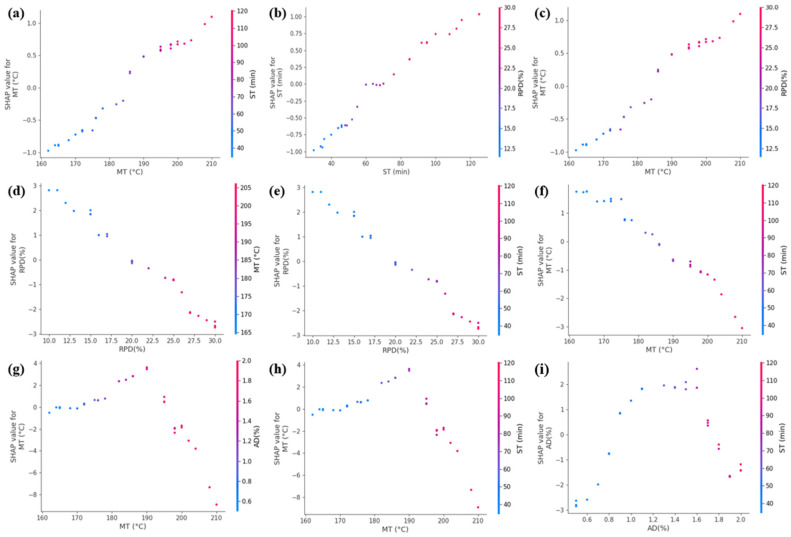
SHAP interaction dependency analysis: (**a**) The interaction between MT and ST on the rutting factor; (**b**) The interaction between ST and RPD on the rutting factor; (**c**) The interaction between MT and RPD on the rutting factor; (**d**) The interaction between RPD and MT on the ductility; (**e**) The interaction between RPD and ST on the ductility; (**f**) The interaction between MT and ST on the ductility; (**g**) The interaction between MT and AD on the residual penetration ratio; (**h**) The interaction between MT and ST on the residual penetration ratio; (**i**) The interaction between AD and ST on the residual penetration ratio.

Further SHAP interaction dependency analysis was conducted to investigate the synergistic effects between the top two influential features for each performance metric (Rutting factor, Ductility, and Residual penetration ratio), as illustrated in Figure 18a–i. For rutting factor (Figure 18a–c), the interaction between MT, ST, and RPD demonstrates a mutually reinforcing relationship—higher values of these parameters collectively enhance high-temperature performance through improved rubber-bitumen interfacial bonding and crosslinking density. In contrast, ductility analysis (Figure 18d–f) reveals an antagonistic pattern. The residual penetration ratio evaluation (Figure 18g–i) uncovers a temperature-mediated dual-regulation mechanism. Moderate MT levels combined with optimal anti-aging agent dosage synergistically inhibit oxidative aging. The methodology not only quantifies nonlinear couplings but also provides actionable guidelines for balancing competing performance requirements in rubber asphalt design.

While SHAP analysis provides valuable interpretability for our machine learning models, several limitations specific to our study should be acknowledged. The relatively limited sample size may affect the stability of SHAP value estimates, particularly for detecting interaction effects. Additionally, the computational demands of Kernel SHAP constrained our analysis to pairwise interactions, potentially missing more complex higher-order relationships. Despite these constraints, SHAP analysis remains the most suitable method for interpreting the complex relationships in our study, with future work planned to address these limitations through expanded datasets and model-specific explainers.

### 3.3. Multi-Objective Optimization

**Figure 19:**
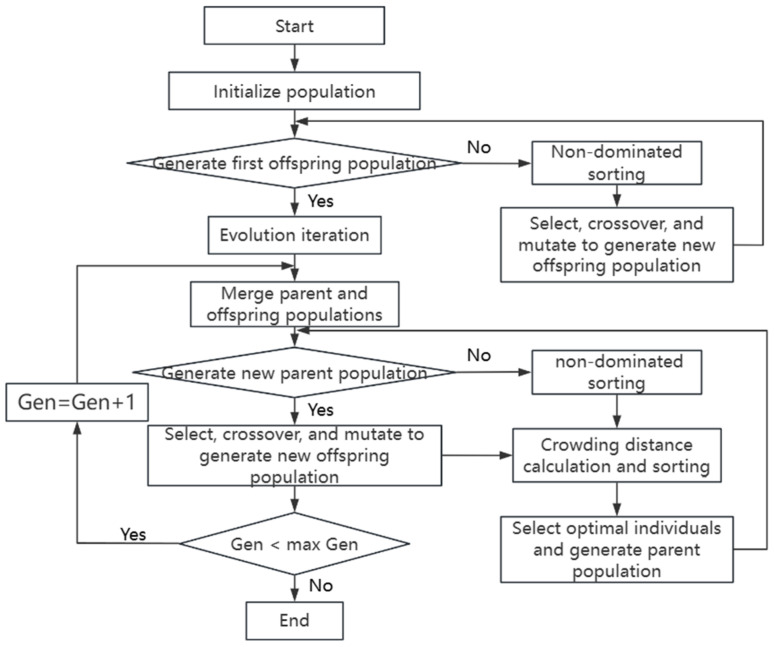
NSGA-II multi-objective optimization algorithm.

Based on the rutting factor, ductility, and residual penetration ratio models established earlier using the optimized BP model, the NSGA-II algorithm was employed to perform multi-objective optimization of the process parameters [49]. Building upon the first-generation genetic algorithm, the NSGA-II algorithm incorporates the concepts of non-dominated sorting and crowding distance, ensuring the final solution set is distributed along the Pareto optimal front. Simultaneously, the introduction of an elitist strategy guarantees that the best individuals are preserved throughout the population evolution process, significantly enhancing the optimization efficiency of the algorithm, as illustrated in Figure 19. The NSGA-II algorithm was configured with the following parameters: a population size of 500 evolved over 500 generations, using simulated binary crossover with a probability of 0.9 and distribution index of 20, and polynomial mutation with a probability of 0.2 and distribution index of 20.

In order to achieve the best high and low temperature performance and anti-aging performance of anti-aging rubber asphalt, the objective function is:

$$f_{1}=\max\left[R_{fi}\left(x_{1i},y_{i},z_{i},w_{i},v_{i}\right)\right]$$

$$f_{2}=\max\left[D_{i}\left(x_{i},y_{i},z_{i},w_{i},v_{i}\right)\right]$$

$$f_{3}=\max\left[R_{pi}\left(x_{i},y_{i},z_{i},w_{i},v_{i}\right)\right]$$

where *x*, *y*, *z*, *w*, *v* stand for RPD, RPM, AD, MT, and ST, respectively.

The constraints are:

$$s.t.\left\{\begin{array}{c} x\leq 30\\ y\leq 100\\ 160\ll w\leq 200 \end{array}\right.$$

**Figure 20:**
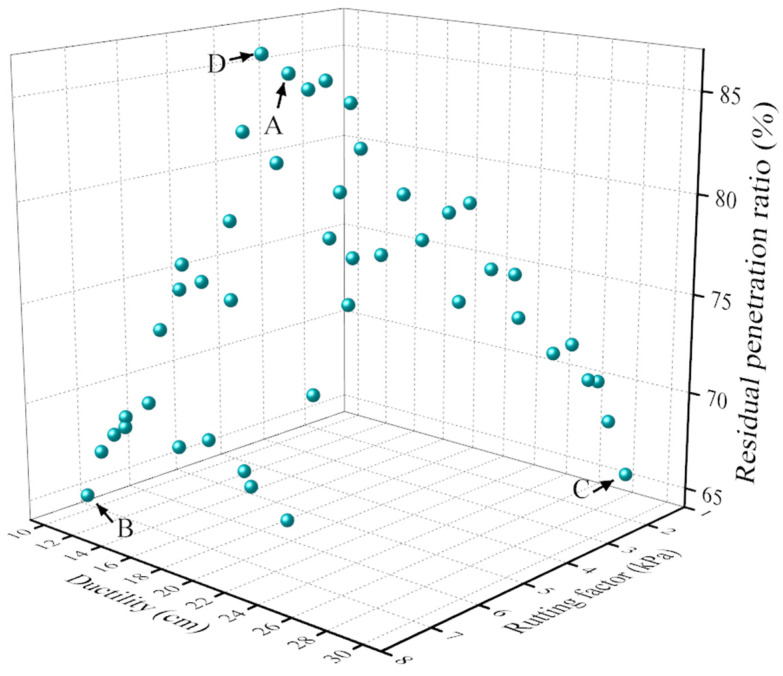
Pareto Solution Sets for Objective Optimization.

Under this setup, the optimization process yielded a Pareto front comprising 50 non-dominated solutions, as illustrated in Figure 20. For the selection of optimal solutions, we employed the ideal point method based on the following procedure: (1) establishing the ideal solution vector composed of optimal values for all three objectives; (2) calculating Euclidean distances between each Pareto solution and the ideal point; (3) identifying solutions that minimize these distances for different application scenarios. From this set, the most suitable parameter combinations were selected for further evaluation based on engineering applicability.

Each point in the Pareto plot from the multi-objective optimization represents a non-dominated solution with respect to the defined objectives, meaning no solution is strictly superior to another. Consequently, all solutions on the Pareto front are considered optimal, and the most appropriate one should be selected based on specific project requirements. This study employed the ideal point method to obtain the final solution under three scenarios: Point A represents the optimal balanced solution combining the rutting factor, ductility, and residual penetration ratio of the modified asphalt. Point B represents the optimal solution for achieving the best rutting resistance of the modified asphalt, without considering other performance aspects. Point C represents the solution maximizing the ductility of the modified asphalt. Point D represents the solution offering the best aging resistance.

To validate the solutions obtained from the NSGA-II multi-objective optimization results proposed in this study, experimental validation was conducted on these optimized solutions. The predicted versus experimental results of the modified asphalt corresponding to Points A, B, C, and D in the Pareto solution set are shown in Table 1. The predicted performance calculated by the NSGA-II solution shows a small difference from the actual results, demonstrating the accuracy and feasibility of the prediction method. The experimental results derived from the Pareto solution set reveal that all error values fall within 10%, which is within the acceptable range generally considered in the field.

**Figure 21:**
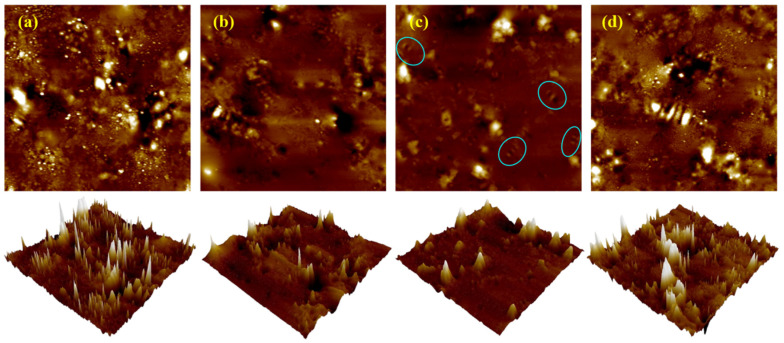
2D and 3D microscopic topography: (**a**) Point A; (**b**) Point B; (**c**) Point C; (**d**) Point D.

**Figure 22:**
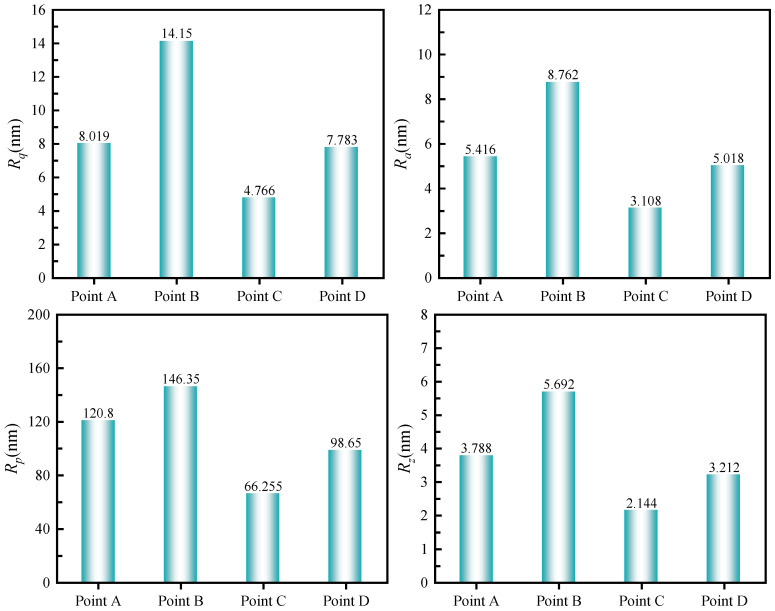
Roughness analysis results.

AFM was further used to observe the microscopic morphology of the modified bitumen under the above four sets of process parameter conditions, and the results are shown in Figure 21, as well as the corresponding roughness analysis results in Figure 22.

Figure 21 demonstrates distinct microscopic morphologies of rubber-modified asphalt under four processing conditions, primarily governed by the rubber particle-asphalt interface state and phase-separated structure. It is worth noting that the rubber powder modified asphalt is different from the matrix asphalt, the rubber powder particles absorbed a large number of light components and waxes in the asphalt, resulting in a reduction in waxes that can be used for crystallization and the formation of the bee-like structure, thus inhibiting the formation of the bee-like structure [48]. The formation of obvious raised particles and continuous interfacial bright ring in the samples of No. A and D, which endowed the modified asphalt with good aging resistance, and the asphalt phase with a weak bee-like structure [50]; the sample of No. B, which was subjected to high temperature and After intense shear, the asphalt phase bee-like structure almost disappeared due to the large absorption of light components, and the interface was damaged due to high-temperature aging, the roughness increased, and the high-temperature performance was improved; Sample No. C retained the phase separation due to the small amount of gum powder doping and oil absorption, so there was a complete bee-like structure (see circle in Figure 21c) in the morphology and the roughness was minimized, which was conducive to the ductility, but the weak interfacial bonding resulted in the low rutting factor.

### 3.4. Discussion

The superior performance of CPO-BP over the standard BP and DBO-BP models can be attributed to its unique multi-modal optimization mechanism. Unlike the gradient-dependent BP, which is prone to local minima, or the DBO with its fixed-radius contraction that may struggle with discrete parameter effects, the CPO algorithm dynamically switches between defensive strategies based on the “predator distance” (analogous to the distance to the optimum). This enables a more adaptive balance between global exploration and local exploitation, leading to a more optimal set of initial weights and biases for the BP network. This finding is consistent with the claims made in the original proposal of the CPO algorithm, which highlighted its effectiveness in overcoming premature convergence [43,44]. When benchmarked against models from prior studies, our CPO-BP model demonstrates highly competitive performance. In contrast, our framework provides a unified and highly accurate predictive tool for three competing objectives simultaneously, addressing a critical gap in multi-objective performance prediction for anti-aging CRMA.

The SHAP analysis provides deep mechanistic insights into the preparation-process-property relationships. The identification of MT as the most dominant and dual-role factor is particularly noteworthy. Its positive correlation with the rutting factor aligns perfectly with the well-established understanding that elevated temperatures promote the devulcanization of crumb rubber and enhance the absorption of light asphalt components, leading to the formation of a more robust, cross-linked rubber-asphalt network that resists permanent deformation. The negative impact of high MT on low-temperature ductility, as revealed by SHAP, provides a quantitative explanation. This degradation is likely due to the excessive breakdown of the rubber’s own elastic polymer network at high temperatures, compromising its flexibility.

Furthermore, the SHAP interaction plots offer the synergistic and antagonistic effects between parameters. The strong positive interaction between MT and ST on the rutting factor indicates that the benefits of high temperature are fully realized only when sufficient time is provided for microstructural development—a nuance often overlooked in conventional single-variable analyses. Similarly, the coupling between RPD and MT on ductility highlights that simply increasing rubber content does not guarantee better low-temperature performance; it must be processed at a moderate temperature to preserve the rubber’s elastic properties.

The NSGA-II multi-objective optimization provided practical solutions for engineering applications. The validity of these optimized solutions was further corroborated by AFM analysis, which revealed distinct microstructural features—such as interfacial morphology and the suppression of “bee-like” structures—that directly correspond to the enhanced macroscopic performance. This micro-macro correlation provides physical evidence supporting the data-driven optimization approach.

Beyond the technical performance, the proposed data-driven framework presents advantages in terms of scalability, cost-effectiveness, and sustainability, which are crucial for its industrial viability. From a scalability perspective, the established predictive models can be rapidly deployed to optimize new formulations, drastically reducing the time- and resource-intensive trial-and-error experiments traditionally required for material development. Economically, while the incorporation of anti-aging agents and the high-shear process may incur initial costs, the enhanced aging resistance directly translates to extended service life and reduced maintenance frequency for asphalt pavements, offering superior long-term economic benefits. From a sustainability standpoint, this study promotes the high-value utilization of waste tires, addressing a critical solid waste problem. The improved durability of the pavement material further contributes to resource conservation and a reduction in the carbon footprint associated with frequent road repairs and reconstructions. Therefore, the proposed approach aligns well with the principles of green engineering and sustainable infrastructure development.

## 4. Conclusions

This study establishes an integrated framework for performance prediction and multi-objective optimization of anti-aging rubber asphalt, yielding the following principal scientific insights:

(1) The hybrid modeling approach combining bio-inspired optimizers with neural networks effectively addresses high-dimensional nonlinear challenges in asphalt performance prediction. The CPO-BP model demonstrates particular advantage through its dynamic balance of global exploration and local refinement.

(2) Mechanistic interpretation through SHAP analysis uncovers the con-text-dependent roles of preparation parameters, particularly highlighting mixing temperature as a dual-function regulator: its elevation improves rutting resistance through enhanced interfacial bonding but concurrently degrades low-temperature ductility by disrupting rubber’s elastic network.

(3) Multi-objective optimization successfully reconciles the inherent trade-offs between competing performance objectives. The derived Pareto-optimal solutions establish that balanced high-low temperature performance with superior aging resistance can be achieved through specific parameter combinations that optimize interfacial development while preserving rubber elasticity.

(4) Despite the promising results, this study has certain limitations that should be acknowledged. The primary constraints include the relatively limited sample size of the experimental dataset and the use of crumb rubber from a single source, which may affect the model’s generalizability to other rubber types and broader application scenarios. Future research will focus on expanding the dataset with more comprehensive experimental designs, incorporating various types of waste rubber materials, and exploring the transferability of the proposed model to different asphalt-rubber systems.

## Figures and Tables

**Table 1:** Predicted and tested values corresponding to the optimal points in the Pareto solution set.

Index		A	B	C	D
RPD (%)		24.36	28.98	16.33	24.36
RPM (mesh)		50.95	75.85	88.31	52.70
AD (%)		1.16	1.72	0.14	1.59
MT (℃)		180.81	194.90	160.02	187.97
ST (min)		74.17	132.52	25.83	74.17
NSGA-Ⅱ	Rutting factor (kPa)	5.29	7.49	1.81	5.49
	Ductility (cm)	17.91	11.3	28.21	16.86
	Residual penetration ratio (%)	86.06	65.42	68.51	86.94
Test Value	Rutting factor (kPa)	5.6	7.33	1.85	5.55
	Ductility (cm)	18.32	11.48	26.33	17.2
	Residual penetration ratio (%)	85.14	66.28	70.22	88.3
Error	Rutting factor	5.94%	2.14%	2.03%	1.09%
	Ductility	2.18%	1.59%	6.07%	2.02%
	Residual penetration ratio	1.12%	1.19%	2.46%	1.56%

## Data Availability

The original contributions presented in the study are included in the article/Supplementary Material, further inquiries can be directed to the corresponding author.

## Supplementary Materials

The following supporting information can be downloaded at https://www.mdpi.com/article/10.3390/ma18235292/s1: Table S1. Differences between CPO, DBO and previous optimization algorithms. Table S2. Technical specifications of asphalt. Table S3. Technical specifications of rubber powder. Table S4. Partial sample data. Table S5. Sample dataset analysis results. Table S6. Performance evaluation of different machine learning prediction models. References [51,52,53] are cited in the supplementary materials.

## Nomenclature

BP	Backpropagation
CPO	Crested Porcupine Optimizer
DBO	Dung Beetle Optimizer
SHAP	Shapley Additive Explanations
CRMA	Crumb Rubber-Modified Asphalt
GA	Genetic Algorithm
PSO	Particle Swarm Optimization
GPR	Gaussian Process Regression
RPD	Rubber Powder Dosage
RPM	Rubber Powder Mesh size
AD	Anti-aging Agent Dosage
MT	Mixing Temperature
ST	Shearing Time
MAE	Mean Absolute Error
RMSE	Root Mean Square Error
MAPE	Mean Absolute Percentage Error
NSGA-II	Non-dominated Sorting Genetic Algorithm II
SVM	Support Vector Machine
ET	Extremely Randomized Trees
KR	Kernel Regression
ANN	Artificial Neural Network
RNN	Recurrent Neural Network
RSM	Response Surface Methodology
AFM	Atomic Force Microscopy

## References

[1] Tian T., Jiang Y.J., Yi Y., Zhang Y., Fan J.T., Zhang W.H. (2024). Dynamic viscoelastic properties of ultra-large particle size asphalt mixture based on fractional derivative constitutive model. Constr. Build. Mater..

[2] Li X.Z., Sha A.M., Jiao W.X., Cao Y.S., Song R.M. (2025). Strain response and creep behavior of asphalt mixture based on multi-damage fractional visco-elasto-plastic constitutive model. Constr. Build. Mater..

[3] Bueno M., Luong J., Terán F., Viñuela U., Paje S.E. (2014). Macrotexture influence on vibrational mechanisms of the tyre-road noise of an asphalt rubber pavement. Int. J. Pavement Eng..

[4] Liu Y., Su P.F., Li M.M., You Z.P., Zhao M.H. (2020). Review on evolution and evaluation of asphalt pavement structures and materials. J. Traffic Transp. Eng. Engl. Ed..

[5] Tian T., Jiang Y.J., Yi Y., Nie C.L. (2025). The splitting fatigue properties of ultra-large particle size asphalt mixture under the coupling effect of temperature and load. Eng. Fract. Mech..

[6] Li Q.S., Zhang H.L., Sun C.J., Li J.C., Feng J.R. (2025). Effect of novel warm-mix compound on road performance and aging resistance of SBR-modified bituminous mixture. Constr. Build. Mater..

[7] Sánchez D.B., Caro S., Santos M.C. (2024). Long-term effectiveness of vegetable-based rejuvenation agents across different aging stages of asphalt pavements. Int. J. Pavement Eng..

[8] Zhang M.M., Su Q.D., Li G.Y., Cao D.W., Yao Y.C., Yang S.Q., Wang S.F. (2025). Enhancing Reutilization of Waste Tires and Sustainability of Environment: Analysis of the Performance and Emission Reduction Mechanism of High Content Rubber Modified Asphalt. Chem. Eng. J..

[9] Chen R.P., Chen L., Hu P., Zhu H.Z., Ou L. (2025). Viscoelasticity stage evolution characteristics and multiphase microstructure of aged crumb rubber-modified asphalt. Constr. Build. Mater..

[10] Gui W.M., Wang L., Li C., Zhan Y., Zhang F. (2025). Effects of aging behavior on the adhesion and low-temperature performance of surfactant-crumb rubber modified asphalt binder. Int. J. Pavement Eng..

[11] He L., Tang J.K., Rong H.L., Xie J., Wang J.P., Wei L. (2025). Recycling of scrap steel slag and waste rubber in asphalt mixtures: Evaluation of road performance and environmental impact analysis. Road Mater. Pavement Des..

[12] Xue Y.H., Zhang H.G., Ge D.D., Lv S.T., Ju Z.H. (2025). The interaction mechanism of activated crumb rubber modified asphalt during the preparation process. Int. J. Pavement Eng..

[13] Liu S., Lu P., Sun X.C., Wang H.C., Fei Z. (2025). Research on the Durability of Modified Crumb Rubber Asphalt Mixtures in High-Altitude and Seasonally Frozen Regions. Coatings.

[14] Zhou Y.X., Xu G., Yu H., Fan Y.L., Wang H.Z., Yang J., Wu Y., Wang H.P., Huang W. (2024). Toughness modification of SBS/CRMA on epoxy asphalt: Curing behaviour and low-temperature cracking characteristic analysis. Int. J. Pavement Eng..

[15] Cao L.P., Su Z.B., Liu R.R., Zhou T. (2022). Optimized formulation of asphalt compound containing bio-oil and shredded rubber. J. Clean. Prod..

[16] He H.Q., Gou P.F., Li R., Pei J.Z., Xie B.W., Yang K. (2022). Optimum preparation and rheological properties of liquid rubber modified asphalt binder. Constr. Build. Mater..

[17] Zhang H.G., Zhang Y.P., Chen J., Liu W.C., Wang W.S. (2024). Influence of preparation parameters on rheological properties and relation analysis of waste rubber modified bitumen mastic. Front. Mater..

[18] Krishna G.G., Jinesh N. (2025). Synergistic interaction effects of process parameters on surface finish in two-way AFM of SS446. Int. J. Interact. Des. Manuf..

[19] Zhang Y., Jiang Y.J., Li C., Bai C.F., Zhang F.X., Li J.X., Guo M.Y. (2025). Prediction of cement-stabilized recycled concrete aggregate properties by CNN-LSTM incorporating attention mechanism. Mater. Today Commun..

[20] Chu Y.H., Zhang Y.P., Li S.Y., Ma Y.G., Yang S.J. (2024). A machine learning approach for identifying vertical temperature gradient in steel-concrete composite beam under solar radiation. Adv. Eng. Softw..

[21] Zhang H.T., Fu X.X., Jiang H.Y., Liu X., Lv L.H. (2015). The relationships between asphalt ageing in lab and field based on the neural network. Road Mater. Pavement Des..

[22] Luo Y.H., Guo P., Gao J.F., Chen Y.Z., Zhou D.J., Hu J.Y. (2023). Prediction and evaluation the moisture damage resistance of rejuvenated asphalt mixtures based on neural network. Constr. Build. Mater..

[23] Ma X.Y., Ma X.J., Wang Z.L., Song S.L., Sheng Y.P. (2023). Investigation of changing SARA and fatigue properties of asphalt bitumen under ageing and analysis of their relation based upon the BP neural network. Constr. Build. Mater..

[24] Rezoug A., Bader-El-Den M., Boughaci D. (2024). Two-step optimization algorithm operated by heuristic and machine learning methods. Discret. Math. Algorithms Appl..

[25] Singh S.K., Kumar M., Singh J. (2023). Integration of Particle Swarm Optimization (PSO) and Machine Learning to Improve Classification Accuracy During Antenna Design. Trans. Electr. Electron. Mater..

[26] Ding N., Ruan X., Wang H., Liu Y. (2025). Automobile Insurance Fraud Detection Based on PSO-XGBoost Model and Interpretable Machine Learning Method. Insur. Math. Econ..

[27] Zhang Y., Zhu B.L., Li J., Wang Q., Hu K.H. (2024). Experimental and machine learning study on mechanical properties and frost resistance of nano-TiO_2_ modified steel fiber reinforced concrete. J. Build. Eng..

[28] Tian K.M., Kang Z.H., Kang Z.J. (2024). A Productivity Prediction Method of Fracture-Vuggy Reservoirs Based on the PSO-BP Neural Network. Energies.

[29] You G.D., Chang Z.C., Li X.Y., Li Z.F., Xiao Z.Y., Lu Y.R., Zhao S.L. (2024). Using enhanced Variational Modal Decomposition and Dung Beetle Optimization Algorithm optimization-kernel Extreme Learning Machine model to forecast short-term wind power. Electr. Power Syst. Res..

[30] Zhang C.P., Liu H.M., Peng Y.M., Ding W.Y., Cao J. (2024). Intelligent Prediction and Application Research on Soft Rock Tunnel Deformation Based on the ICPO-LSTM Model. Buildings.

[31] Xia H.Z., Chen L.M., Xu H.W. (2025). Multi-strategy dung beetle optimizer for global optimization and feature selection. Int. J. Mach. Learn. Cybern..

[32] Wang R., Salleh H., Li K., Abdul-Samad Z. (2024). A conceptual cost estimation model for building construction projects by hybrid Back-Propagation Neural Network and Dung Beetle Optimizer algorithm. J. Asian Arch. Build. Eng..

[33] (2023). Standard Test Method for Determining the Rheological Properties of Asphalt Binder Using a Dynamic Shear Rheometer.

[34] (2017). Standard Test Method for Ductility of Asphalt Materials.

[35] (2022). Standard Test Method for Effect of Heat and Air on a Moving Film of Asphalt Binder (Rolling Thin-Film Oven Test).

[36] (2017). Standard Test Method for Penetration of Bituminous Materials.

[37] Xue Y.Y., Ge D.D., Lv S.T., Duan D.F., Deng Y.J. (2023). Evaluation of asphalt modified with bio-oil and high rubber content: Low temperature and short mixing time production condition. Constr. Build. Mater..

[38] Zhang J.W., Chen M.Z., Wu S.P., Zhou X.X., Zhao G.Y., Zhao Y.C., Cheng M. (2021). Evaluation of VOCs inhibited effects and rheological properties of asphalt with high-content waste rubber powder. Constr. Build. Mater..

[39] Jamal M., Giustozzi F. (2022). Chemo-rheological Investigation on Waste Rubber-Modified Bitumen Response to Various Blending Factors. Int. J. Pavement Res. Technol..

[40] Sernas O., Cygas D., Vaitkus A., Gumauskait V. The Influence of Crumb Rubber on Modified Bitumen Properties. Proceedings of the 10th International Conference on Environmental Engineering (ICEE).

[41] Li H.A. Application of BP neural network in logistics forecasting. Proceedings of the 1st International Conference on Modelling and Simulation.

[42] Quan R., Zhou Y.L., Yao S.Y., Wan H., Chang Y.F. (2025). Improving the performance of a polygonal automobile exhaust thermoelectric generator with a crested porcupine optimizer. Appl. Therm. Eng..

[43] Lei W.L., Gu Y.F., Huang J.Y. (2024). An Enhanced Crowned Porcupine Optimization Algorithm Based on Multiple Improvement Strategies. Appl. Sci..

[44] Abdel-Basset M., Mohamed R., Abouhawwash M. (2023). Crested Porcupine Optimizer: A new nature-inspired metaheuristic. Knowl. Based Syst..

[45] Li C.J., Li S., Xie Y., Wu J.Z., Zhang C.Y. (2025). Optimization of laser welding process parameters considering carbon emissions and weld quality based on DBO-BP and NSGA-II. Int. J. Precis. Eng. Manuf. Technol..

[46] Wang Y., Shi S., Mai S.P. (2025). Composite high order super-twisting sliding mode control algorithm for PMSMs based on dung beetle optimization. J. Power Electron..

[47] Dong A.Q., Liu L.J., Zhao C.C., Guan Y. (2025). Neural Network Prediction of Locomotive Engine Parameters Based on the Dung Beetle Optimization Algorithm and Multi-Objective Optimization of Engine Operating Parameters. Sensors.

[48] Sun E.Y., Zhao Y.Q., Wang G.Z. (2024). Nano surface evolution properties of crumb rubber modified asphalt due to aging and adhesion failure mechanism. Constr. Build. Mater..

[49] Zhao Z.B., Liu B., Zhang C.R., Liu H.R. (2019). An improved adaptive NSGA-II with multi-population algorithm. Appl. Intell..

[50] Sun E., Zhao Y.Q., Cai R. (2024). Characterization of microstructural evolution of asphalt due to water damage using atomic force microscopy. Constr. Build. Mater..

[51] Jegatheesan N., Ibrahim M.R., Ahmed A.N., Koting S., El-Shafie A., Katman H.Y.B. (2024). Modeling the properties of terminal blend crumb rubber modified bitumen with crosslinking additives. Constr. Build. Mater..

[52] Uwanuakwa I.D., Ali S.I.A., Hasan M.R.M., Akpinar P., Sani A., Shariff K.A. (2020). Artificial Intelligence Prediction of Rutting and Fatigue Parameters in Modified Asphalt Binders. Appl. Sci..

[53] Al-Sabaeei A.M., Alhussian H., Abdulkadir S.J., Sutanto M., Alrashydah E., Mabrouk G., Bilema M., Milad A., Abdulrahman H. (2023). Computational modelling for predicting rheological properties of composite modified asphalt binders. Case Stud. Constr. Mater..

