# Supplementary material for: Performance Prediction and Process Optimization of Aging-Resistant Rubber-Modified Asphalt via Enhanced BP Neural Network and Multi-Objective NSGA-II
Source: Materials (Basel). 2025 Nov 24;18(23):5292. doi: 10.3390/ma18235292 (PMC12692807; doi:10.3390/ma18235292)
Supplement: Supplementary file 1 [file materials-18-05292-s001.zip › materials-3975519-SI.pdf]

Table S1. Differences between CPO, DBO and previous optimization algorithms.

Optimization Algorithm	Key Advantages	Limitations in multi-objective prediction
PSO	Fast convergence, simple implementation	Prone to local optima; limited exploration in high-dimensional, multi-objective spaces
GA	Strong global search capability	High computational cost, slow convergence; inefficient in handling strongly coupled objectives
GPR	Provides uncertainty estimation	Poor scalability with large, high-dimensional datasets; complex modeling of multi-output problems
CPO	Dynamic global-local balance via multi-modal defense strategies; Superior escape from local optima	Requires validation
DBO	Efficient search via rolling, breeding, and foraging behaviors	Requires validation

Table S2. Technical specifications of asphalt.

Test Item		Measured Value	Specification Requirement
Penetration at 25℃ (0.1mm)		64	60～80
Penetration Index		-1.05	-1.5～+1.0
Ductility at 10℃(cm)		28	≥20
Softening Point (℃)		48.5	≥46
Dynamic Viscosity at 60℃（Pa·s)		220	≥180
Wax Content（%）		1.84	≤2.0
Flash Point (℃)		275	≥260
Solubility (%)		99.93	≥99.5
After RTFOT Aging	Mass Loss (%)	-0.14	≤±0.6
	Residual Penetration Ratio (%)	69.7	≥65
	Residual Ductility at 10℃ (cm)	7.4	≥6

Table S3. Technical specifications of rubber powder.

Test Item	Measured Value	Specification Requirement
Relative Density	1.22	1.1~1.3
Carbon Black Content (%)	35	≥28

Ash Content (%)	4.6	≤8
Rubber Hydrocarbon Content (%)	60	≥48
Moisture Content (%)	0.38	＜1

Table S4. Partial sample data.

Number	RPD (%)	RPM (mesh)	AD (%)	MT (℃)	ST (min)	Rutting factor (kPa)	Ductility (cm)	Residual penetration ratio (%)
1	15	60	0.7	170	40	2.8	24.3	76.5
2	20	80	1	180	50	3.9	20.1	82.4
3	25	100	1.5	190	70	5.2	16.8	85.7
4	10	40	0.5	165	35	2.1	26.5	70.2
5	20	60	0.9	175	55	3.5	21.7	80.9
6	20	80	1.3	185	65	4.7	18.3	85.1
7	30	100	1.8	195	85	6.5	14.6	73.8
8	15	60	0.7	170	40	2.7	24.6	76.3
9	20	80	1.1	180	55	4	19.6	83.2
10	10	40	0.3	155	25	1.5	29.2	64.7
…	…		…	…		…	…	…

Table S5. Sample dataset analysis results.

Feature types	Maximum value	Minimum value	Mean value	Standard deviation
RPD (%)	30.0	10.0	21.3	6.2
RPM (mesh)	100.0	40.0	75.2	22.5
AD (%)	2.0	0.3	1.3	0.5
MT (°C)	215.0	155.0	184.9	14.6
ST (min)	135.0	25.0	70.1	28.1
Rutting factor (kPa)	8.8	1.5	4.9	2.0
Ductility (cm)	29.2	7.9	18.6	5.4
Residual penetration ratio (%)	88.7	51.2	76.1	8.6

Table S6. Performance evaluation of different machine learning prediction models.

Ref.	(ML) models	RMSE	R^2
Current study	BP	0.27	0.957
	CPO-BP	0.15	0.993
	DBO-BP	0.26	0.985
Jegatheesan[51]	Tree	0.44	0.822
	SVM	0.11	0.989
	ET	0.46	0.805
	KR	0.32	0.905
	GPR	0.19	0.966
Uwanuakwa[52]	ANN	1.2	0.96
	GPR	1.0	0.97
	RNN	0.8	0.96
	SVM	1.1	0.95
Sabaeei[53]	RSM	-	0.97
	GPR	-	0.99

Tree: Decision Tree; SVM: Support Vector Machine; ET: Extremely Randomized Trees; KR: Kernel Regression; GPR: Gaussian Process Regression; ANN: Artificial Neural Network; RNN: Recurrent Neural Network; RSM: Response Surface Methodology.

[51] Jegatheesan, N.; Ibrahim, M.R.; Ahmed, A.N.; Koting, S.; El-Shafie, A.; Katman, H.Y.B. Modeling the properties of terminal blend crumb rubber modified bitumen with crosslinking additives. *Constr. Build. Mater.* **2024**, *444*, 137648.

[52] Uwanuakwa, I.D.; Ali, S.I.A.; Hasan, M.R.M.; Akpinar, P.; Sani, A.; Shariff, K.A. Artificial Intelligence Prediction of Rutting and Fatigue Parameters in Modified Asphalt Binders. *Appl. Sci.* **2020**, *10*, 7764.

[53] Al-Sabaeei, A.M.; Alhussian, H.; Abdulkadir, S.J.; Sutanto, M.; Alrashydah, E.; Mabrouk, G.; Bilema, M.; Milad, A.; Abdulrahman, H. Computational modelling for predicting rheological properties of composite modified asphalt binders. *Case Stud. Constr. Mater.* **2023**, *19*, e02651.
